# Three-dimensional culture suppresses pancreatic fibroblast activation and limits paracrine signaling between pancreatic cancer cells and fibroblasts

**DOI:** 10.1016/j.bbrep.2026.102843

**Published:** 2026-10-27

**Authors:** Nino Javakhishvili, Anais Zourelidis, Bogusz Trojanowicz, Yoshiaki Sunami, Jörg Kleeff

**Affiliations:** a Department of Visceral, Vascular and Endocrine Surgery, Martin-Luther-University Halle-Wittenberg, University Medical Center Halle, Halle (Saale), 06120, Germany; b Institute of Medical and Public Health Research, Ilia State University, Tbilisi, 0162, Georgia

**Keywords:** Pancreatic ductal adenocarcinoma (PDAC), Pancreatic fibroblasts, 3D magnetic levitation culture

## Abstract

**Background:**

Pancreatic fibroblasts play a significant role in the development, progression, and treatment limitations of pancreatic ductal adenocarcinoma (PDAC), making them an essential area of research. Traditional 2D in vitro models fail to fully capture complexities of these stromal cells in PDAC such as interactions, morphology, and response to stimuli present in *in vivo* environments, making 3D culture systems a promising platform for further studies.

**Methods:**

We compared the behavior of pancreatic fibroblasts in 2D and 3D culture systems, by analyzing the effects of different cultures on transcriptomics. Primary murine pancreatic fibroblasts were cultured in monolayers and 3D spheroids. An indirect co-culture system between fibroblasts and KC murine pancreatic cancer cells (KCs) served to investigate paracrine effects of cancer cells. RNA sequencing and RT-PCR were utilized for data analysis, supported by Oil Red O staining.

**Results:**

Inactivation of fibroblasts in the 3D magnetic levitation model was noted by downregulation of activation markers *Acta2, Fap,* and *Ctgf*, and upregulation of a lipid-droplet stabilizer *Plin2*. The HIF-1 signaling pathway was enriched in the 3D model compared to 2D. Oil Red O staining demonstrated upregulation of cytoplasmic lipid droplets, in 3D vs. 2D culture models. Activation marker downregulation and lipid accumulation seen in both monocultures and co-cultures in 3D indicated a cell-specific behavior rather than tumor cell influence.

**Conclusion:**

The 3D magnetic levitation culture could serve as a model to study fibroblasts in an inactivated state, while co-culture with KC tumor cells could be helpful for cancer-fibroblast paracrine interaction studies.

## Highlights

• Pancreatic tissue fibroblasts in 3D magnetic spheroids display reduced activation compared to 2D cultures.
• 3D pancreatic tissue fibroblast spheroids reveal hypoxia and ferroptosis enrichment compared to 2D models.
• 2D pancreatic tissue fibroblasts show upregulation of focal adhesion and ECM-receptor interactions.
• 3D pancreatic tissue fibroblast spheroids limit paracrine effects with pancreatic cancer cells compared to 2D cell cultures.

## 1 Introduction

Pancreatic ductal adenocarcinoma (PDAC), despite the relatively rare incidence rates, is an extremely aggressive malignancy. According to Global Cancer Statistics 2024, PDAC is the eleventh most common cancer while having the sixth highest morality rate responsible for around one in twenty cancer deaths in both males and females combined [2]. The increasing incidence is especially prominent in Western countries [3]. U.*S. cancer* statistics 2025 reported it as the third leading cause of cancer-related deaths with a 5-year survival rate as low as 8% [4]. Pancreatic cancer is being predicted to rise as the third leading cause of cancer deaths in the European Union surpassing breast cancer [5]. Considering the statistics, significant progress is necessary to combat the disease course and improve the outcomes.

Multiple features define PDAC biology one of them being a pronounced stromal development, associated with pancreatic fibroblasts. Although they make up less than 5% of pancreatic cells, fibroblasts play essential roles in tissue homeostasis [6], [7]. They help to maintain the basement membrane and tissue structure [8] and regulate ECM turnover by secreting matrix metalloproteinases (MMPs) and their inhibitors – tissue inhibitors of metalloproteinases (TIMPs). When injured, the pancreas triggers pancreatic fibroblasts activation, leading to increased ECM production for tissue repair. In vitro, they can switch between quiescent and activated states [9], [10]. Fibroblasts are recruited early during carcinogenesis, and they can influence cancer invasiveness and metastasis by interacting with tumor cells through direct cell contact as well as by paracrine communication. Pancreatic fibroblasts in the context of pancreatic cancer are also termed cancer-associated fibroblasts (CAFs) and they contribute to abundant desmoplasia development in PDAC [11]. In their quiescent state, fibroblasts store lipids and vitamin A in perinuclear droplets and express markers such as Vimentin and glial fibrillary acidic protein (GFAP). Upon activation – typically due to tissue damage – these lipid droplets disappear, cells extend cytoplasmic processes and adopt a myofibroblastic phenotype marked by alpha smooth muscle actin (ACTA2) expression [9]. *Acta2* is one of several key genes contributing to PDAC phenotype. Alpha smooth muscle actin (ACTA2) is a stromal marker in pancreatic adenocarcinoma, with higher expression in fibroblasts and the level indirectly proportional to patient survival. The fibroblasts expressing ACTA2 are more activated contributing to the aggressiveness of pancreatic cancer by enhancing invasion, proliferation and colony formation [12], [13]. Connective tissue growth factor (CTGF), is another profibrotic growth factor significantly upregulated in pancreatic cancer, especially in stroma, promoting ECM development, tumor proliferation and aggressive desmoplastic reaction [14], [15]. Fibroblast Activation Protein (FAP) is a key marker of a specialized subset of cancer-associated fibroblasts (CAFs) serving not only as a marker but also as an active mediator of immune evasion and therapy resistance and FAP-positive CAFs contribute to remodeling the extracellular matrix [16], [17].

2D culture has been the most actively used model in preclinical research and has provided significant data for pancreatic cancer pathogenesis. The model is associated with a short time of culture formation, high performance and reproducibility, and cost-effectivity. Yet it is also associated with many limitations including drastically different cell morphology and metabolism as compared to 3D cultures. Cells in 2D are unable to mimic the structures of tumors therefore creating disturbances in cellular and environmental interactions and negatively affecting studies trying to understand the mechanisms of tumor and stromal development and interactions [18], [19]. 2D systems are also described to alter gene expression as well as mRNA splicing and metabolism of cells [20], [21], [22], [23]. Cells growing in 2D monolayers are also unable to represent the tumor conditions accurately, having unlimited access to nutrients and oxygen unlike tumors developing and growing *in vivo*. 3D culture systems aim to overcome some disadvantages of traditional 2D models and help prevent over- and underestimation of drug effect studies [18]. They were also shown to be much more similar to cells growing *in vivo* regarding transcriptomics, metabolism, and cell signaling [24], [25]. Several well-established three-dimensional (3D) culture models are currently used in cancer research, with spheroids and organoids representing two of the most widely employed approaches. Spheroids are typically generated from established cell lines or primary cells through spontaneous or induced cell aggregation. Compared with conventional two-dimensional (2D) cultures, spheroids more closely recapitulate key features of the tumor microenvironment, including increased cell–cell interactions, altered intercellular signaling, and the formation of oxygen and nutrient gradients [19]. Organoids, in contrast, are self-organizing 3D structures derived from stem cells, primary tissues, or tumor specimens and can reproduce aspects of the cellular heterogeneity and architecture of the tissue of origin. Both spheroid- and organoid-based systems can be adapted to co-culture approaches, allowing the investigation of interactions between tumor cells and different components of the tumor microenvironment. In particular, spheroid-based co-culture models have been widely employed to study tumor–stromal interactions under more physiologically relevant conditions [26], [27], [28].

We have previously described the transcriptome changes in pancreatic fibroblasts between standard 2D and inverse models co-cultured with mouse pancreatic cancer cells [29]. The results demonstrated strong susceptibility of fibroblasts to cancer cell signaling depending on the developed model, with increased transcriptome changes with closer proximity. Here, we are presenting the transcriptomic differences between pancreatic fibroblasts in 2D cultures and spheroids formed by magnetic nanoparticle assembly, as well as their transcriptomic alterations in KC co-cultures. The magnetic levitation-based 3D culture system, first introduced by Souza and colleagues in 2010, combines the benefits of carrier-free systems without the shear stress of agitation. It also supports larger spheroids and greater cell numbers than the hanging drop method. This model relies on cells incubation with gold and magnetic iron oxide (MIO) nanoparticles, the combination of which creates a magnetizable matrix as well as a magnetic lid placed above the culture leading to levitation of the magnetized cell-hydrogel complexes at the air-liquid interface and aggregation into 3D structures [30]. At proper concentrations, these particles are non-toxic and do not affect proliferation [31]. This study model since then has been used for multiple other research projects, including for solid tumor studies [32], [33].

Our project explored transcriptomic variation between pancreatic fibroblasts in 2D and 3D magnetic levitation and analyzed paracrine effects through indirect co-culture model with murine pancreatic cancer cells (KC).

## 2 Materials and methods

### 2.1 Mouse models

All mouse experiments were approved by the Martin-Luther University Halle-Wittenberg (Nr. K2aM3) and conducted in accordance to the European Parliament 2010/63/EU and ARRIVE guidelines. Pancreatic fibroblasts were isolated from pancreata of 7-10-week-old wild-type C57BL/6 J mice obtained from Charles River Laboratories (Sulzfeld, Germany).

### 2.2 Isolation and culture of primary pancreatic fibroblasts

Primary pancreatic fibroblast isolation was performed as we previously described [29] using Nycodenz gradient centrifugation protocol as developed by Apte et al. [34] with minimal modifications. The 7-10-week-old wild-type C57BL/6J mice pancreata were obtained after euthanization done by cervical dislocation. Each isolated pool 4-10 pancreata for equal gender ratio to balance out any effects of sex. Dissected pancreata were injected with an enzyme solution containing 10% collagenase P (Roche, Germany, (cn) 11213857001), 10% pronase (Roche, Germany, (cn) 11459643001); and 1% DNase (Machnery-Nagel, Germany, 740963). The mixture was filtered, washed with HBSS and centrifuged mixture pellet was resuspended with 4.75 mL GBSS with 0.3% BSA and 4 mL of 28.7% solution of Nycodenz (Alere Technologies AS, Norway, (cn) 1002424) in GBSS without NaCl. After addition of 3 mL GBSS with 0.3% BSA and centrifugation, the pellet was resuspended with DMEM low glucose medium with 40% Nutrient Mixture F12, 16% FBS, 1% Amphotericin B, and 1% Penicillin/Streptomycin.

### 2.3 Isolation and culture of primary pancreatic cancer cells (KCs)

Primary pancreatic cancer cell line, generated from a KC (*Ptf1a/p48-Cre; lox-stop-lox-Kras*^*G12D/+*^) mouse was provided by Dr. Bo Kong (University of Heidelberg). The tumor cell isolation protocol was previously published [35]. Cell authenticity was confirmed by PCR and Sanger sequencing demonstrating Kras G12D mutation and wild-type p53 status (Table 1).

## 3 Method details

### 3.1 Cell culture

Primary pancreatic fibroblasts were cultured in DMEM low glucose medium (Sigma-Aldrich, Germany, (cn) D5546) supplemented with 40% Nutrient Mixture F12 (Gibco, UK, (cn) N6658), 16% FBS (Sigma-Aldrich, Germany, (cn) S0615), 1% Amphotericin B (Sigma-Aldrich, Germany (cn) A2942), and 1% Penicillin/Streptomycin (Gibco, UK, (cn) 15140122) initially after isolation. A few days later, the medium was changed to the same composition medium but without Amphotericin B. The KC cells were cultured in DMEM high glucose medium (Sigma-Aldrich, Germany (cn) D5796) supplemented with 1% Penicillin/Streptomycin and 10% FBS. KC cells used for experiments were between the first and fifteenth passages. Both pancreatic fibroblasts and KC cells were cultured at 37 °C in 5% CO2. The cells were routinely tested to rule out Mycoplasma contamination.

### 3.2 3D magnetic culture and co-culture models

Our study involved co-culture between primary murine pancreatic fibroblasts and KC cells. Two co-culture methods were used, traditional 2D transwell system and 3D model consisting of magnetically levitated pancreatic fibroblasts in the insert with tumor cells at the bottom without direct contact. The co-culture was performed using cell culture inserts with polyethylene membrane and a pore size of 1 μm inserted into a well of 6-well culture plates. In the 2D co-culture, pancreatic fibroblasts were grown on top of the membrane insert, with the KCs seeded at the bottom of a 6-well plate. The 3D co-culture model contained the pancreatic fibroblasts magnetically levitated within the insert resulting in quick aggregation and spheroid formation while the tumor cells were seeded at the bottom of the well. The size of the pores of the insert ensuring no cells could pass through and the interaction was only by exchanging of soluble substances (Fig. 1).

The 3D model required a higher number of pancreatic fibroblasts for sufficient RNA yield, 5 × 10^5^, compared to 10^5^ for 2D cultures, for levitation as was determined by preliminary experiments. At time zero, 10^5^ KC cells in 2 mL DMEM Advanced medium were seeded at the bottom of a 6-well plate. 75 μl of Shuttle™-PL nanoparticles were added to passage 1-2 pancreatic fibroblasts once they reached 70-80% confluency in 25 cm^2^ flasks. Cells were incubated with nanoparticles for at least 8 h, nanoparticle uptake was confirmed by light microscopy (Fig. 2). The 3D culture was also accompanied by a control setup where fibroblasts with and without exposure to magnetic nanoparticles were used for bulk RNA sequencing to rule out the effect of nanoparticles on cell culture conditions. After incubation, cells were washed with HBSS, trypsinized, and centrifuged (336g, 23 °C, 10 min). The cell pellet was resuspended in 2 mL DMEM Advanced medium, 5 × 10^5^ pancreatic fibroblasts were added to a 6-well plate with a cell-repellent surface. A magnetic lid was applied to levitate cells, which was observed macroscopically. Pancreatic fibroblasts were levitated for 24 h before experiments. All co-culture experiments were repeated at least three times independently of each other. The cells were counted with a Neubauer counting chamber.

**Fig. 1:**
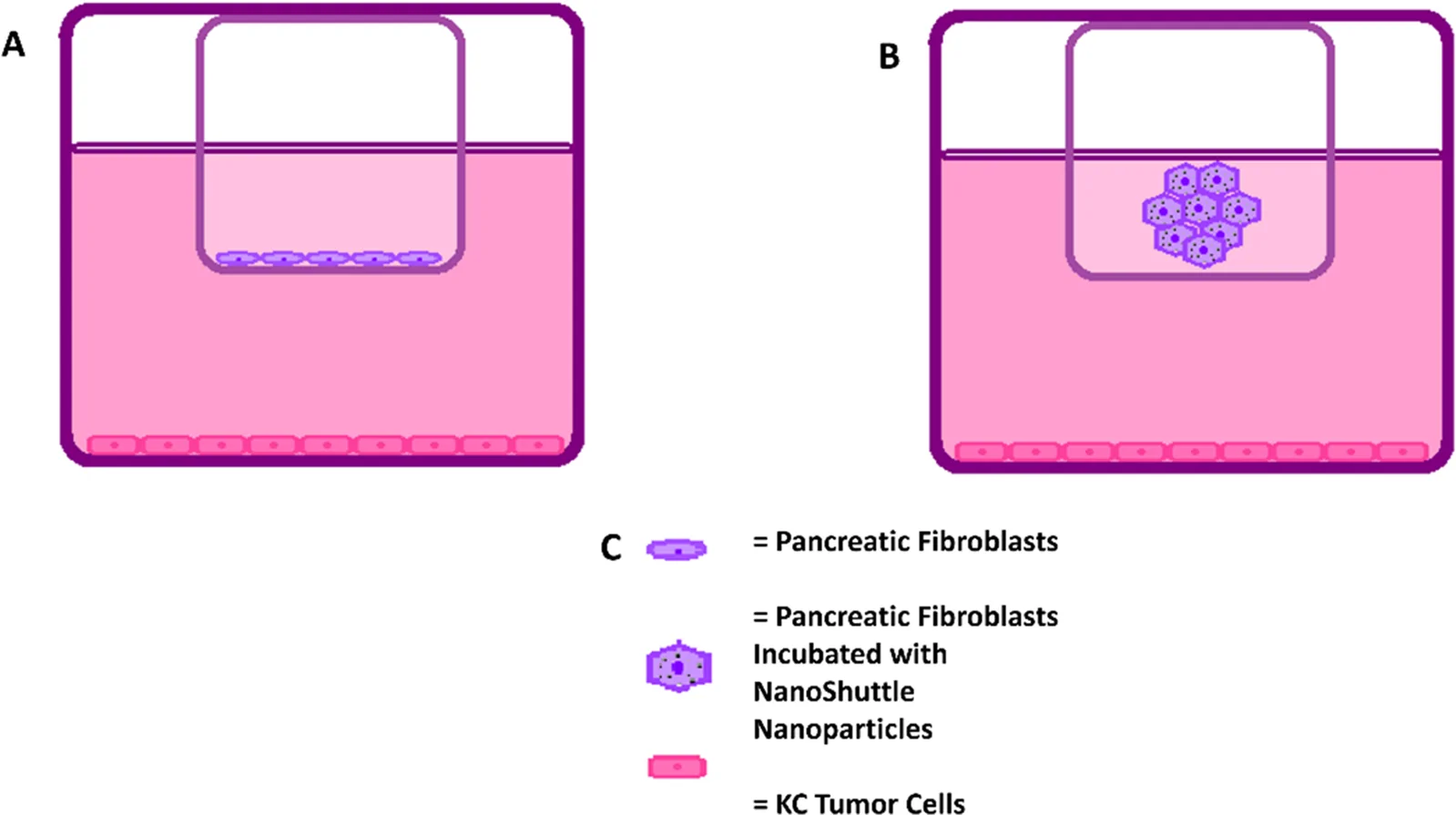
Different co-culture models; Pancreatic fibroblasts within the cell culture insert and tumor cells at the bottom of the well **(A)**; Pancreatic fibroblasts in levitation within the cell culture insert and tumor cells at the bottom of the well **(B)**; Cells used in both models **(C)**.

**Fig. 2:**
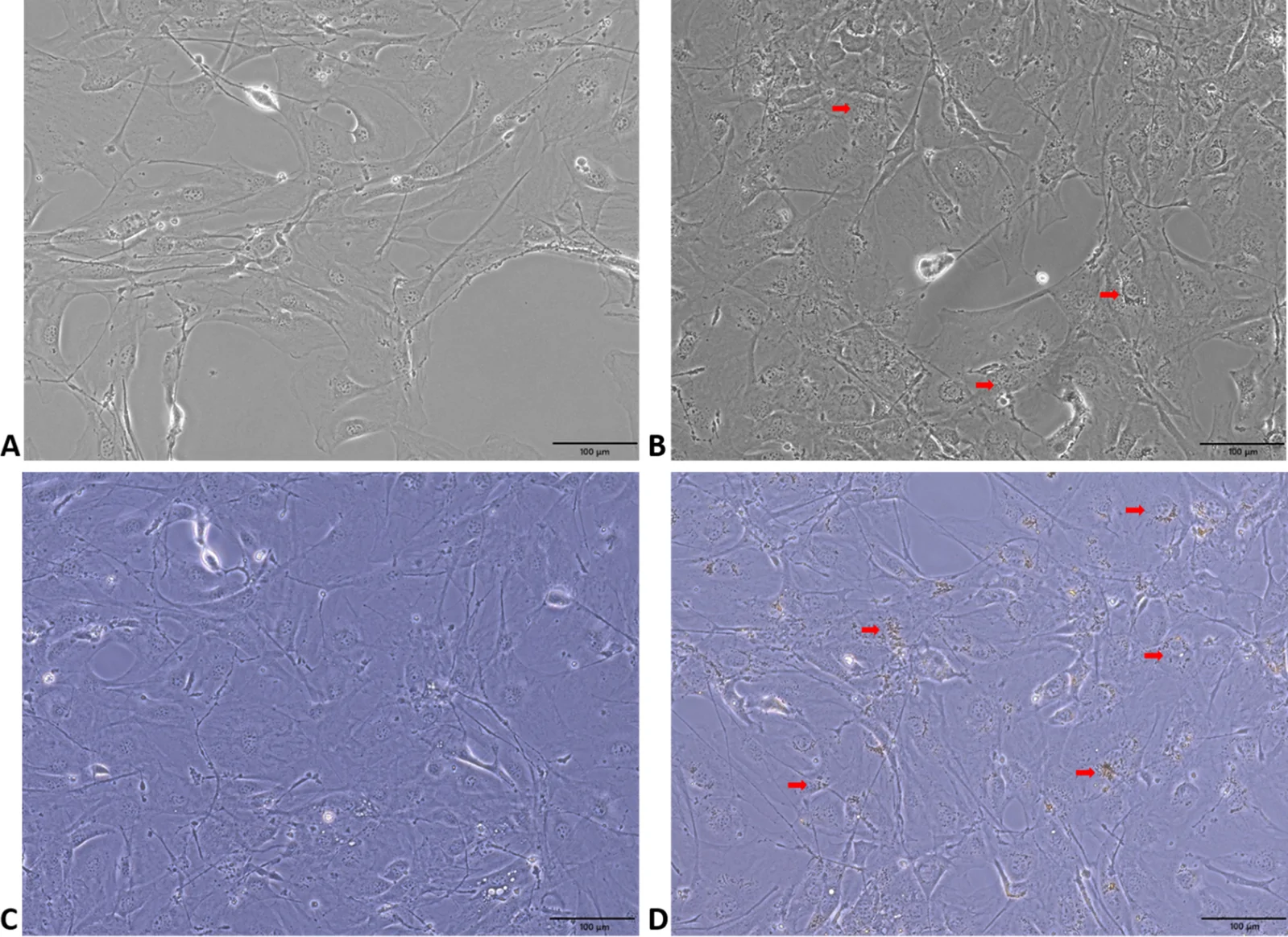
Pancreatic fibroblasts before **(A)** and **(C)** and after **(B)** and **(D)** magnetic nanoparticle addition; Nanoparticles are indicated by red arrows; Phase contrast microscopy in black and white **(A)**, **(B)** and in color setting in **(C)**, **(D)** better visualizing the magnetic nanoparticles; Magnetic nanoparticles are indicated by red arrows.; Scale bar = 100 μm.

### 3.3 Preparation and fixation of spheroids for immunocytochemical staining

Fixation of pancreatic fibroblasts during levitation for immunocytochemical and Oil Red O staining was based on a previously described method used for 3D cardiomyocyte models [36]. For this purpose, the levitated pancreatic fibroblasts were handled by placing 6-well holding drive underneath for the subsequent steps. The cells were washed with 1xPBS, then fixed for 1h in 4% PFA at room temperature (RT) and wash 3 times in 1xPBS. The fixed cells were embedded between two layers of Tissue-Tek® OCT™ Compound and frozen at −20° until further use. The pancreatic fibroblasts were identified by macroscopic inspection, appearing as small brown-to-black dots. Subsequently, Cryostat was used for 10 μm thick sections which were mounted on on SuperFrost™ Plus slides and stored at −20 °C until staining procedures.

### 3.4 Immunocytochemistry of pancreatic fibroblasts in 2D and 3D cultures

Primary pancreatic fibroblasts cells were cultured in DMEM low glucose medium (Sigma-Aldrich, Germany, (cn) D5546) with 40% Nutrient Mixture F12 (Gibco, UK, (cn) N6658), 16% FBS (Sigma-Aldrich, Germany, (cn) S0615), and 1% Penicillin/Streptomycin (Gibco, UK, (cn) 15140122) on a glass slide and incubated for 24 h. Afterwards, the slides were fixed with 4% PFA for 1h at RT. The inoculation and primary fixation of the OT with pancreatic fibroblasts as well as the preparation and fixation of the pancreatic fibroblasts 3D cultures, were performed as described above. The spheroids were circled using a Pap Pen. Slides were washed with PBS 3X and incubated with primary antibodies at 4 °C in a damp chamber overnight. The antibodies were diluted with Antibody Diluent (Agilent, USA, cn SO809) with dilutions of 1:100 anti-alpha-smooth muscle actin antibody (17H19L35, Rabbit-monoclonal antibody, Thermo Scientific, Germany); 1:250 anti-fibroblast activation protein (GTX35261, Gene Tex); 1:500 anti-cytokeratin 19 (SA30-06, Rabbit-monoclonal antibody, Thermo Scientific, Germany). PBS-incubated slides were used as a negative monoculture. The next day slides were briefly washed with PBS and incubated with Dako LSAB2 system-HRP kit (Agilent, USA, cn K0609) secondary antibody, washed with PBS for 15min and incubated with Dako liquid DAB + substrate chromogen system kit (Agilent, USA, cn K3468) according to the manufacturer's instructions. The slides were counterstained with hematoxylin solution (Merck, Germany, cn 105175) diluted 1:5 in distilled water. Slides were covered with aquatex medium (Merck, Germany, cn 108562). The imaging was done with Keyence BZ-X800 (Keyence, Osaka, Japan) brightfield microscope.

### 3.5 Oil red O staining

The oil red o stock solution was prepared by mixing of 300 mg Oil Red O (Sigma-Aldrich, USA) in 100 mL Isopropanol and kept at 4°. The working solution was freshly prepared prior to staining. For Oil Red O staining of 3D pancreatic fibroblasts spheroids, the cryosections were washed in 1x PBS and then washed in freshly filtered and 5:2 distilled water-diluted oil red staining solution. The slides were incubated for 15 min. The slides were washed multiple times with distilled water and counterstained with freshly filtered hematoxylin solution (Merck, Germany) for 45 s. Subsequently, the slides were washed repeatedly with distilled water, covered with aquatex medium (Merck, Germany) and cover slides. The human preadipocyte cell line, SGBS, in an adipocyte differentiated state was used as a positive monoculture for lipid droplet appearance and Oil Red O staining. The imaging was done with Keyence BZ-X800 (Keyence, Osaka, Japan) brightfield microscope.

### 3.6 RNA extraction

RNA extraction from 2D pancreatic fibroblasts in cell culture inserts was performed by washing the inserts with 2 ml HBSS (Gibco, UK, cn 14175095) and adding 2 ml Trypsin–EDTA (0.05%) (Gibco, UK, cn 25300062) for 10 min at 37 °C. Afterwards, the Trypsin was inactivate by 2 ml Advanced DMEM/F-12 medium (Gibco, UK, cn 12634010) medium, the insert was rinsed once more with 1 ml Advanced DMEM/F-12 medium, which was also added to the tube and the suspension was centrifuged at 336 g for 10 min at 23 °C. This was followed by the supernatant being discarded, after this 300 μl Qiazol Lysis Reagent (Qiagen, Hilden, cn 79306) was added to the pellet, and the suspension was vortexed.

Pancreatic fibroblasts in levitation in the cell culture insert were used for RNA isolation after 24 and 72 h time points. Pancreatic fibroblasts had been in levitation for 24 h prior to KC incubation, making the actual isolation time points – 48 and 96h. Pancreatic fibroblasts in levitation within a cell culture insert without tumor cell co-culture or in monoculture were used as controls. The levitating spheroids were pipetted out together with the medium, the cell culture insert was washed three times with 2 mL HBSS each, the suspension was centrifuged at 336g at 23° for 10 min. The supernatant was discarded and 300 μl Qiazol Lysis Reagent (Qiagen, Hilden, (cn) 79306) was added to the pellet.

A direct RNA Microprep Kit (Zymo Research, Germany, (cn) R2061) was used according to the manufacturer's instructions for both 2D and 3D pancreatic fibroblasts RNA isolation. The eluted RNA concentration and quality (A260/A280 ratio) was evaluated with NanoDrop One (Thermo Scientific, Gemany).

### 3.7 RNA sequencing and data analysis

RNA sequencing was performed as we previously described [29]. Library construction, sequencing, as well as bioinformatic analysis, were done by Novogene (UK). Sample integrity and purity were verified with an agarose gel electrophoresis with the Agilent 2100 Bioanalyzer. Novogene NGS RNA Library Prep Set (PT042) was used for library preparation, and their quality control was performed using Qubit 2.0 Fluorometer Bioanalyzer. Afterwards, library quantification with qPCR and size distribution detection with Agilent 2100 was done. Sequencing and paired-end reads (150 bp) creation were done with Illumina NovaSeq 6000. Base calling was done using CASAVA. Raw reads were processed through fastq. GC content distribution and base error were also determined. For mapping the reads to the reference genome, mus musculus (Genom ID ensembl\_mus\_musculus\_grcm38\_p6\_gca\ _000001635\_8), the HISAT2 algorithm (v2.0.5, parameter: –dta, –phred33) was used. Reads were quantified using FeatureCounts (v1.5.0-p3, parameter: default), and fragments per kilobase per million mapped reads (FPKM) of the genes were calculated. FPKM > 1 was set as the threshold for gene expression. DESeq2 R package (v1.20.0, padj < 0.05) was used for differential gene expression analysis. ClusterProfiler (v3.8.1, padj < 0.05) R package was utilized for Gene Ontology (GO)- and Kyoto Encyclopedia of Genes and Genomes (KEGG)-analysis. The data discussed in this manuscript have been deposited in NCBI's Gene Expression Omnibus [1] and are accessible through GEO Series accession number GSE328041 (https://www.ncbi.nlm.nih.gov/geo/query/acc.cgi?acc=GSE328041).

### 3.8 Quantitative RT-PCR

PCR (Rotor-Gene Q, Qiagen) was used to amplify the selected genes using the primers listed in Table 1 and × HOT FIREPol® EvaGreen® qPCR Supermix (Solis BioDyne, Estland, cn 08–36-0000S). For each qPCR sample, a master mix of 4 μl 5 × HOT FIREPol® EvaGreen® qPCR Mix Plus, 0,5 μl antisense primer (10 μM), 0,5 μl sense primer (10 μM), and 11 μl RNase-free water was prepared. All primers (100 μM) were diluted 1:10 with RNase free water. The total volume of each sample was 20ul with 2ul of cDNA added to the described master mix. Each sample was tested in duplicate. The following steps were performed for gene amplification by the Rotor-Gene Q cycler: hold 15 min at 95 °C, 40 cycles of 15 s at 95 °C, 20 s at 60 °C, and 20 s at 72 °C. The raw data was analyzed by Comparative Quantification [37] with primers provided by Metabion.

**Table 1:** Primer sequences used in the study; *Rpl37a*, *Fap*, *Ctgf*, *Acta2*, and *Plin2* for target validation by qPCR; *Kras* and *Trp53* for PCR and Sanger sequencing.

Primer	Amplicon Size	Sequence
*Rpl37a* s as	92 bp	CCAAGATGAAGAGACGAGCCG AGAGGTGGTGTTGTAGGTCCA
*Fap* s as	113 bp	CCGCGTAACACAGGATTCACTG CACACTTCTTGCTCGGAGGAGA
*Ctgf* s as	131 bp	TGCGAAGCTGACCTGGAGGAAA CCGCAGAACTTAGCCCTGTATG
*Acta2* s as	138 bp	TGCTGACAGAGGCACCACTGAA CAGTTGTACGTCCAGAGGCATAG
*Plin2* s as	105 bp	GACAGGATGGAGGAAAGACTGC GGTAGTCGTCACCACATCCTTC
*Kras* s as	163 bp	TGT AAG GCC TGC TGA AAA TGA C TTT ACA AGC GCA CGC AGA C
*Trp53* s as	111 bp	AAG TCA CAG CAC ATGACG GA GGA GGC TGC CAG TCC TAA C

## 4 Quantification and statistical analysis

### 4.1 Statistical analysis

Every experiment was conducted using three biological replicates, and for RNASeq replicates (n = 3) were sequenced individually. The significance of differences in gene expression was determined using parametric t-tests or non-parametric Mann-Whitney Tests (both two tailed), depending on the data distribution False discovery rate was controlled by using p-adjust value with Benjamini and Hochberg's method [38]. A p-adjusted value of <0.05 was deemed statistically significant for gene expression. A KEGG-pathway and a GO-term were also determined to be statistically significantly enriched based on whether or not the p-value was <0.05. For qPCR data evaluation, expression values were analyzed using a lognormal ordinary one-way ANOVA followed by multiple comparisons, based on three biological replicates (n = 3). Results are presented as mean ± standard deviation.

## 5 Results

### 5.1 Immunocytochemistry

Immunocytochemistry was performed on pancreatic fibroblasts prior to experiments using activation markers ACTA2 and FAP and epithelial marker CK-19 to visualize the purity of the isolated cells. The murine pancreatic fibroblasts were strongly positive for ACTA2 and FAP in early and late passages, while retaining negative immunoreactivity for CK-19 Fig. 3.

**Fig. 3:**
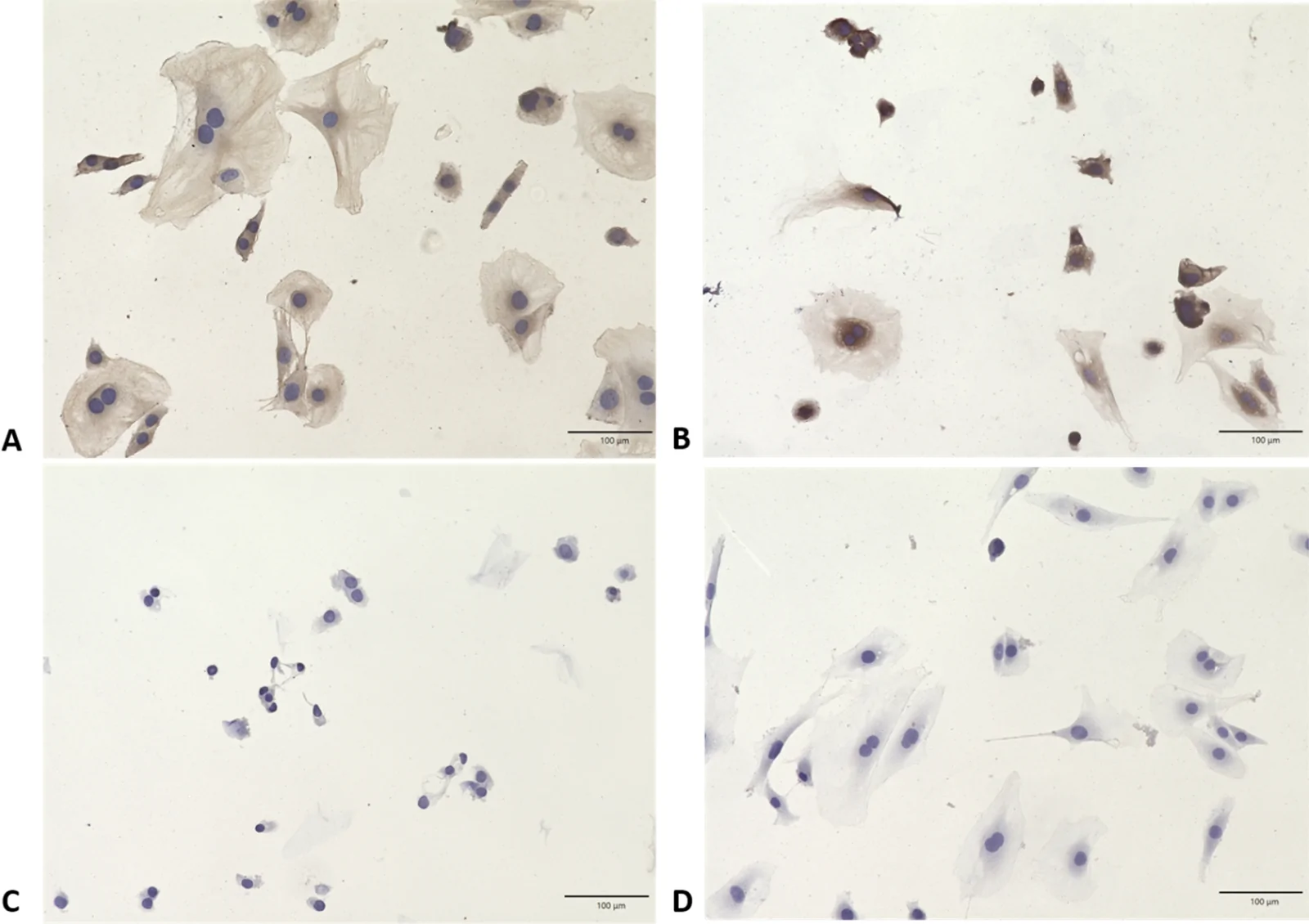
Immunocytochemistry (ICC) performed on 2D pancreatic fibroblasts cells for canonical marker expression validating the purity of murine pancreatic fibroblasts; ACTA2 positive pancreatic fibroblasts **(A)** FAP positive pancreatic fibroblasts **(B**) CK-19 negative pancreatic fibroblasts **(C)** Negative control **(D).** Scale bar = 100 μm.

### 5.2 RNA sequencing data quality, mapping results, and correlation of biological replicates

RNA sequencing was performed at 24h and 72h after isolating pancreatic fibroblasts monocultures as well as pancreatic fibroblasts co-cultured with KCs in 2D and 3D models. The base error rate was <0.03% in all samples. Over 89.80% reads were deemed clean after raw read filtering, and the GC content distribution range was between 52.72% and 50.90% in all the samples.

All samples displayed the quantity of total mapped reads or fragments (TMR) > 90% and <10% of multiple mapped reads (MMR). The reads in the reference genome were mainly exonic (>82.85%) and only minimally intronic or intergenic. Pearson correlation coefficient corresponding to the FPKM of each sample was used to calculate the correlation coefficient between the biological replicates, which ranged between 0.905 and 0.990 (M = 0.964, σ = 0.018) for all biological replicates.

### 5.3 Development of pancreatic fibroblasts in 3D culture over time and 2D model comparison

For initial model comparison, monoculture development and their differential gene expression over time were analyzed. Bar graphs show the number of DEGs developing in each monoculture system between 24 and 72 h. Volcano plots illustrate the numbers, significance, and logarithmic fold changes of differentially expressed genes (DEGs) of pancreatic fibroblasts in 3D and 2D monocultures between 24 and 72 h 2D cultures showed no significant DEGs between the two time points, 3D cultures had 1224 significant DEGs Fig. 4. Dot plots were used for Kyoto Encyclopedia of Genes and Genomes (KEGG)-pathway enrichment analysis due to high number of DEGs being found between the 24h and 72h time points for 3D monocultures. They displayed 18 significantly enriched pathways between the target time points, 7 down- and 11 upregulated Fig. 5. Pathways like ECM-receptor interaction, critical for pancreatic fibrosis and tumor stroma, and focal adhesion, strongly linked to integrin signaling and pancreatic fibroblasts migration and contractility, were upregulated in 24h 3D cultures while Rap1 signaling pathway, regulator of integrin activation and cell adhesion, was upregulated at 72 hTable 2. 14 genes in both ECM-receptor interaction and Focal adhesion pathways are overlapping and upregulated in 24h compared to 72h 3D cultures such as *Vtn*, *Col6a3*, *Itga11*, *Col6a2*, *Col6a1*, *Col1a2*, *Tnxb*, *Lama4*, *Col1a1*, *Thbs3*, *Col4a2*, *Col4a1*, *Lama2*, and *Col4a5* which indicate enhanced extracellular matrix production and cell adhesion. The 72h-enriched Rap1 signaling genes like *Fgf*, *Pdgf*, *Pgf*, *Vegfr*(*Flt4*), *Angptl1* indicate cell proliferation, remodeling or angiogenesis, while Integrin gene such as *Itgb3* and *Itgba2b* point toward stronger cell adhesion and migration.

**Fig. 4:**
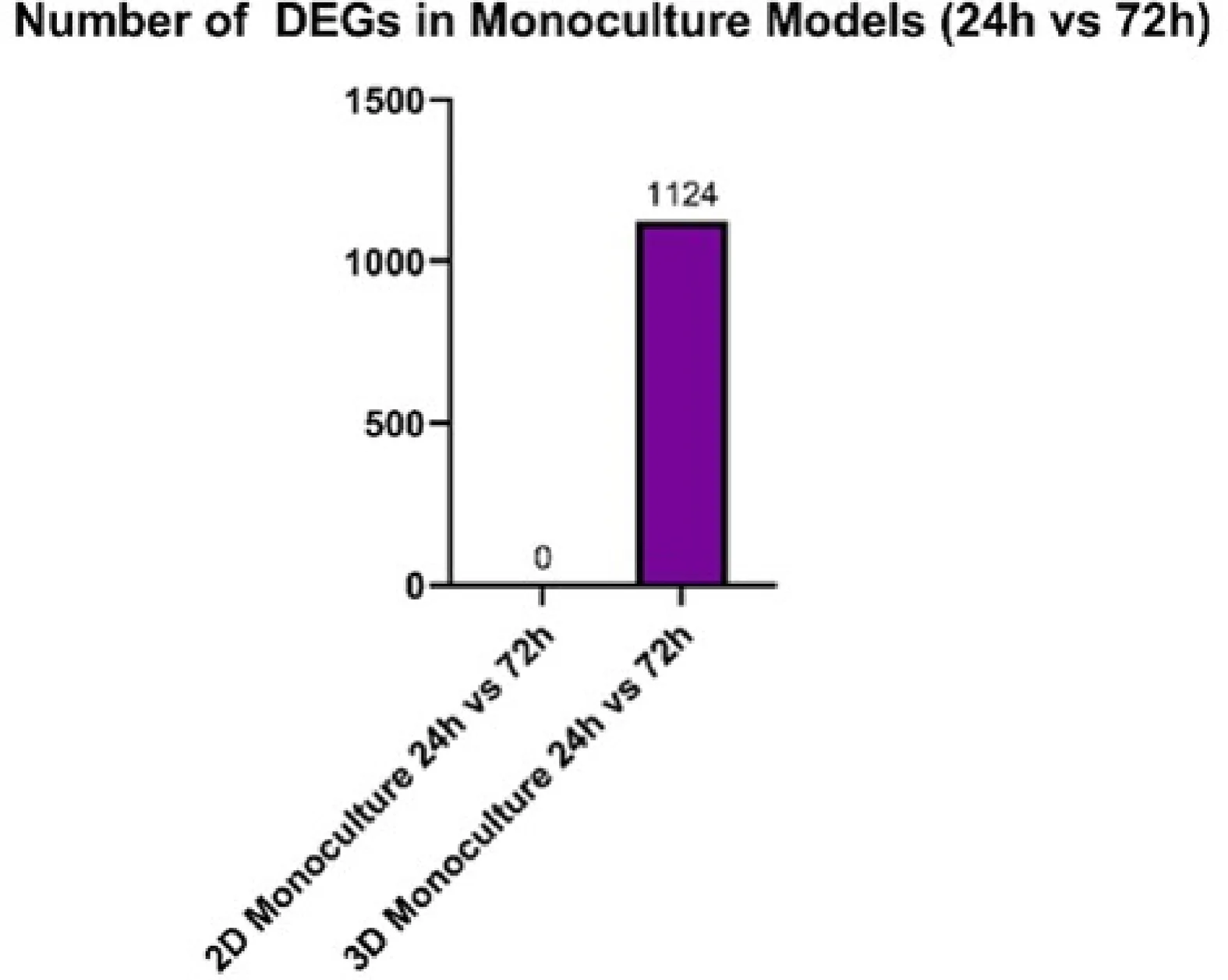
Bar graphs visualizing differential gene expression in 2D and 3D model monocultures between the target time points.

**Fig. 5:**
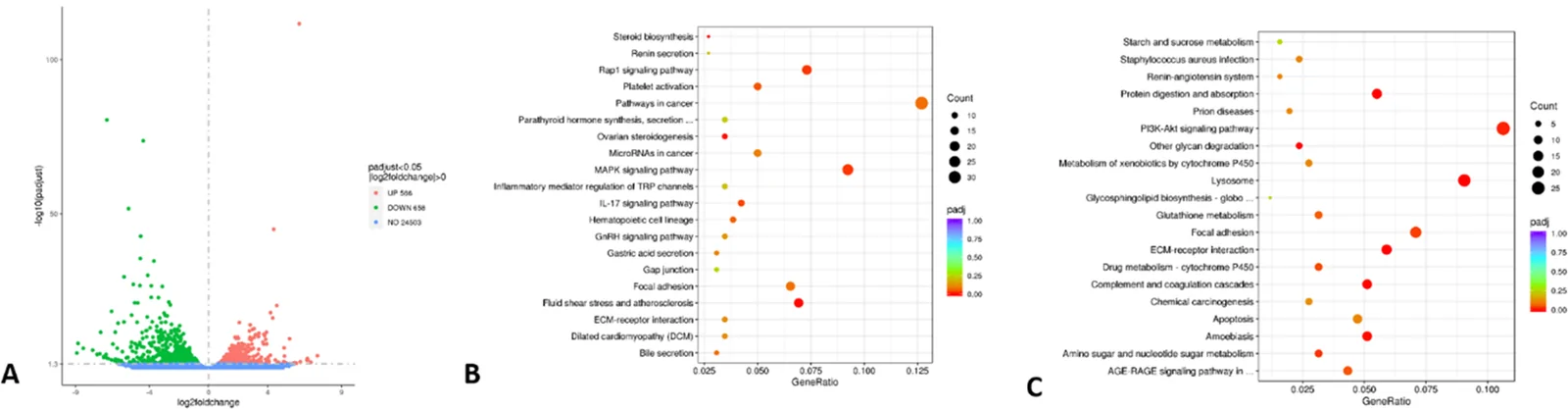
Visualization of differential gene expression and KEGG pathway enrichment between 24 and 72 h in 3D monoculture **(A)** Volcano plot showing DEGs in pancreatic fibroblasts 3D monocultures; **(B)** and **(C)** Dot plots illustrating down- and upregulated KEGG pathway enrichment in 3D monocultures.

**Table 2:** Pathway enrichment analysis.

3D 24h vs 3D 72h monoculture	
**Downregulated**	Steroid biosynthesis Fluid shear stress and atherosclerosis Ovarian steroidogenesis MAPK signaling pathway Rap1 signaling pathway IL-17 signaling pathway Platelet activation
**Upregulated**	Lysosome ECM-receptor interaction Protein digestion and absorption Complement and coagulation cascades Other glycan degradation Amoebiasis PI3K-Akt signaling pathway Amino sugar and nucleotide sugar metabolism Focal adhesion Drug metabolism - cytochrome P450 AGE-RAGE signaling pathway in diabetic complications

Table 2 displays the complete list of KEGG pathways identified as significantly enriched (adjusted p-value &lt; 0.05) in the 3D monoculture at 24h compared to 72h.

The KEGG analysis showed no pathway enrichment in the 2D model monocultures as no DEGs were significantly regulated.

Sequencing on pancreatic fibroblasts exposed to magnetic nanoparticles compared to fibroblasts without magnetic culture did not identify any significant differential gene expression between the groups (**FS1**) consistent with preserved normal fibroblast morphology and proliferation after nanoparticle exposure (Fig. 2).

### 5.4 Differences between 2D and 3D monoculture

To compare the pancreatic fibroblasts behavior in 2D and 3D cultivation, monocultures of both 2D and 3D conditions were compared at 24 and 72h Fig. 6. Volcano plots show the number of significantly differently expressed genes in the comparison of 2D vs. 3D monoculture. Differential gene expression between 2D and 3D monoculture groups showed 1549 upregulated and 1615 downregulated genes at 24h and 2295 upregulated and 2202 downregulated genes at 72h Fig. 7. KEGG enrichment comparison between the monocultures displayed significant variation. 21 pathways were significantly enriched in 2D and 3D monoculture comparisons at 24h and 12 pathways at 72h. 3D monocultures showed consistent upregulation of HIF-1 signaling pathway as well as ferroptosis compared to 2D monocultures in both 24 and 72h data Fig. 8, Table 3, Table 4.

**Fig. 6:**
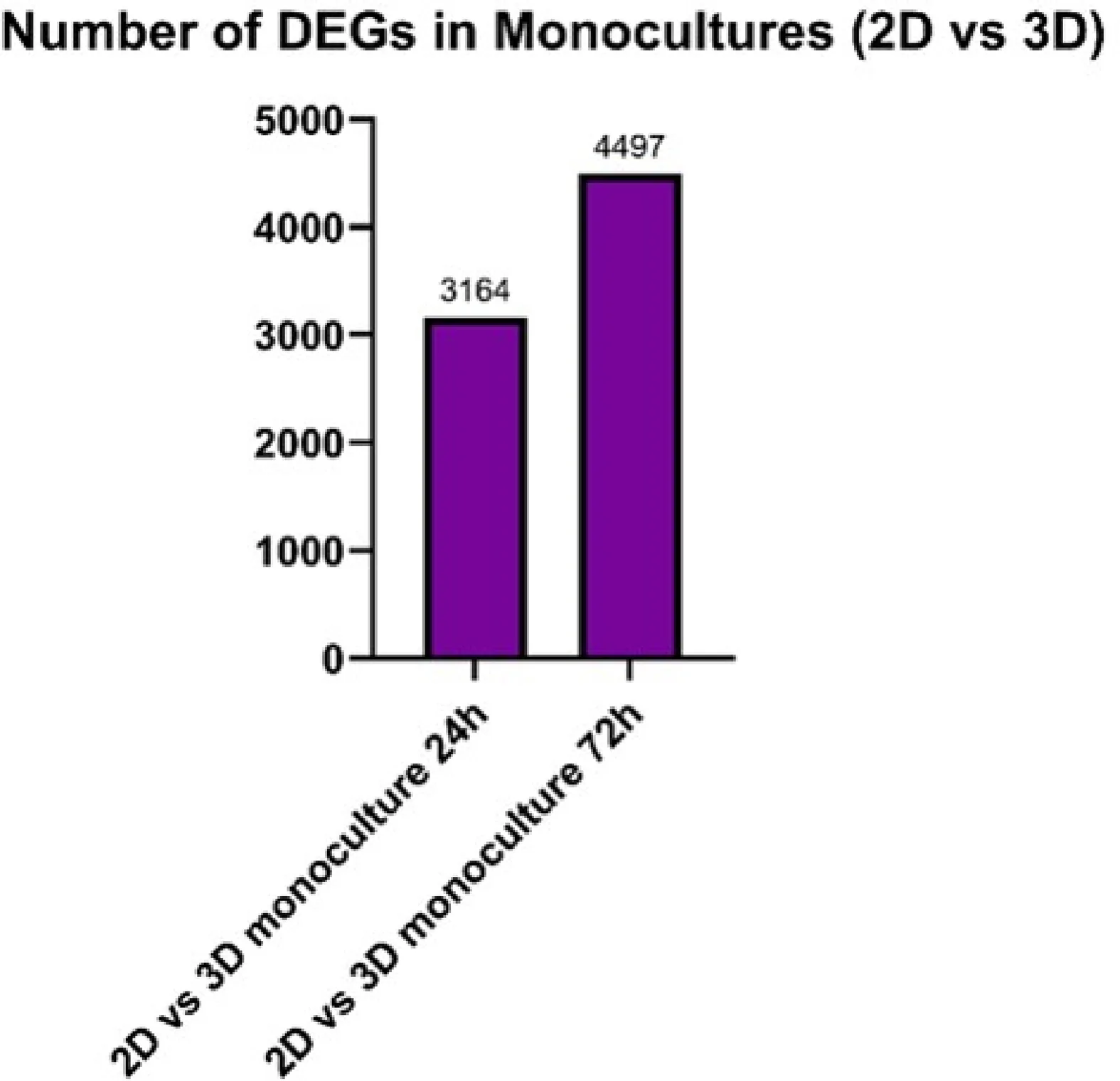
Bar graphs demonstrating differential gene expression between the monoculture groups in 2D and 3D models in 24 and 72 h data.

**Fig. 7:**
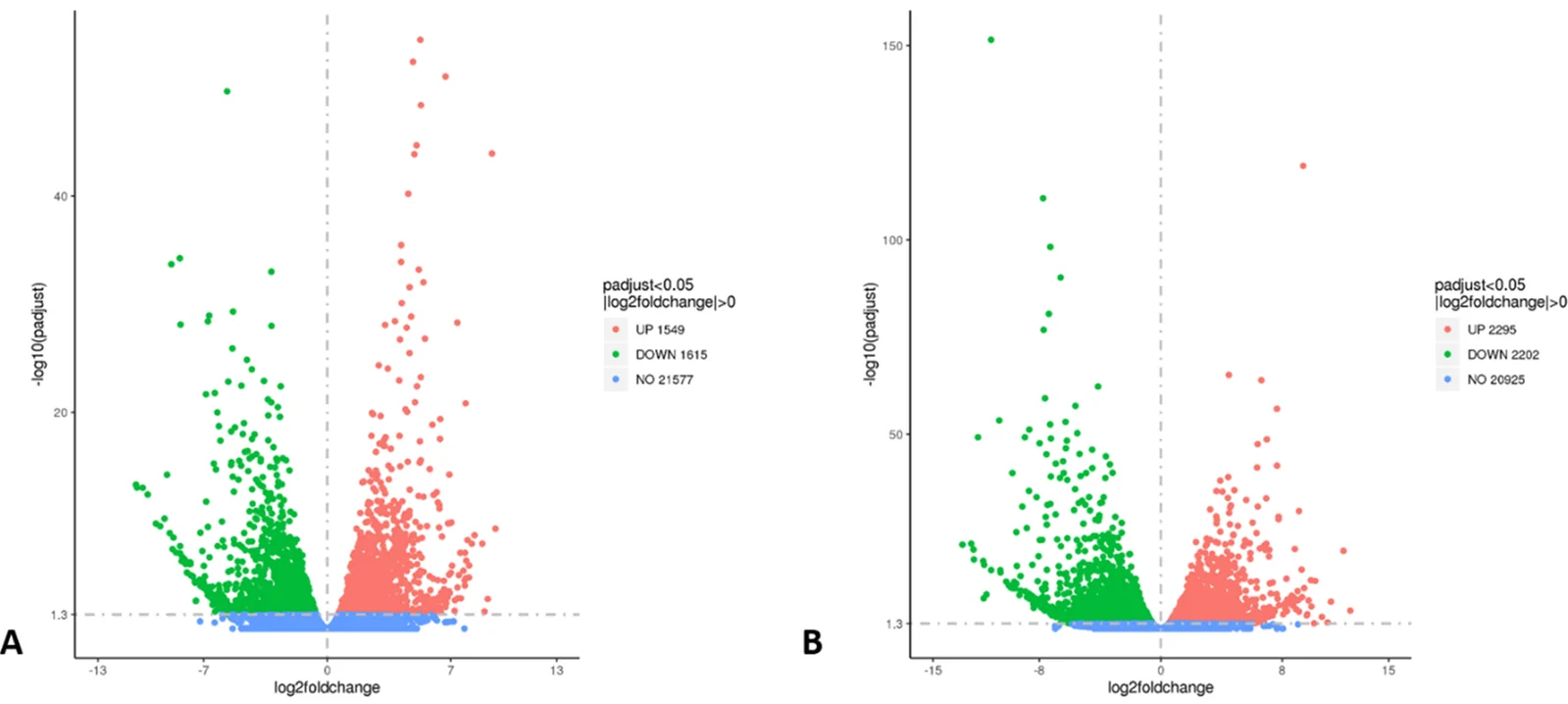
Volcano plots visualizing DEGs between 2D and 3D monocultures; 1549 up- and 1615 downregulated genes between 24h 2D and 3D monoculture groups **(A)**; 2295 up- and 2202 downregulated genes between 72h 2D and 3D monoculture groups **(B)**.

**Fig. 8:**
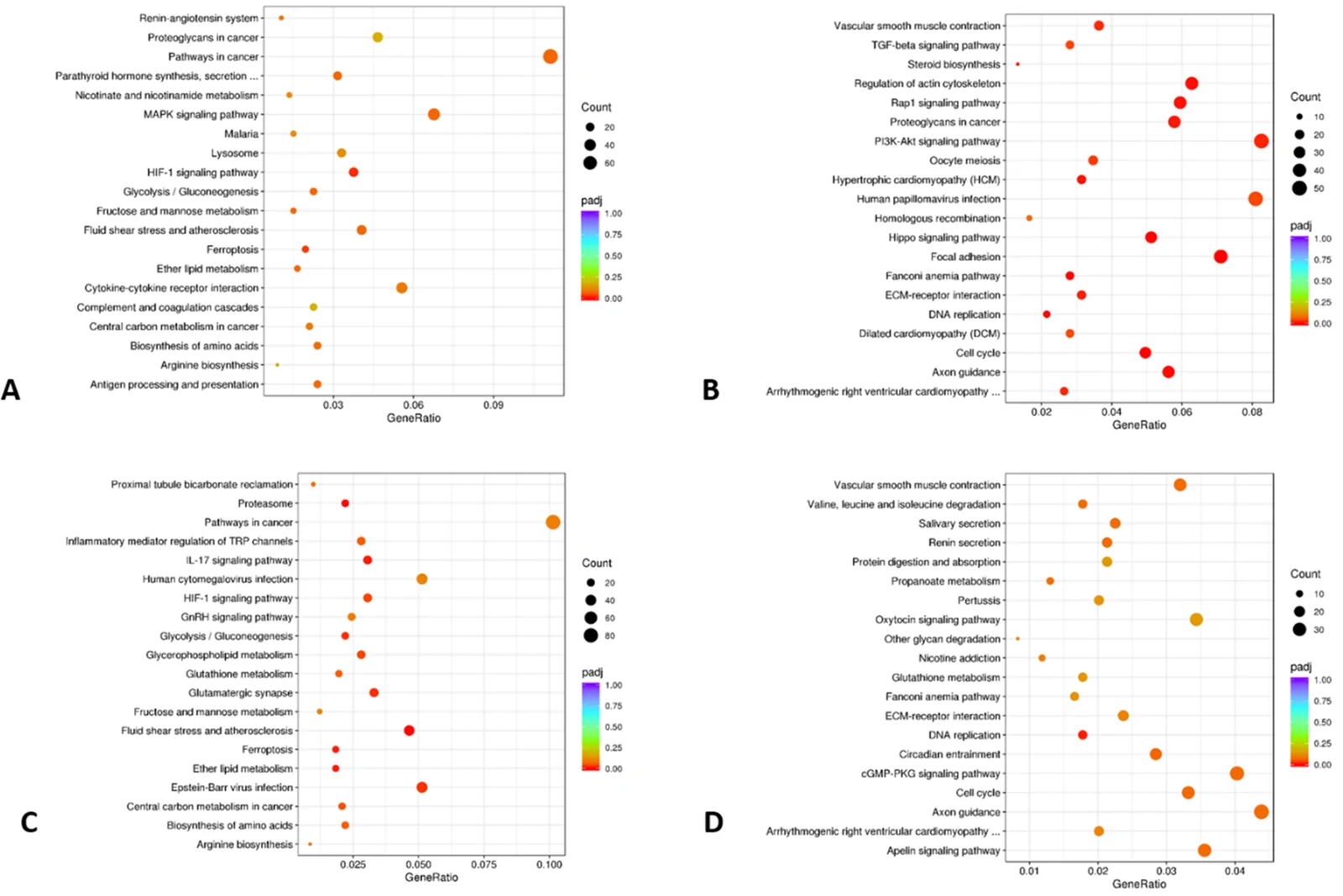
Dot plots for KEGG pathway enrichment demonstrating the regulation between 2D and 3D monocultures; 24h up- and downregulated pathways between 2D and 3D samples **(A)** and **(B)**; 72h up- and downregulated pathways between 2D and 3D samples **(C)** and **(D)**.

**Table 3:** Pathway enrichment analysis.

2D 24h vs 3D 24h Monoculture	
**Downregulated**	HIF-1 signaling pathway Ferroptosis
**Upregulated**	Focal adhesion Cell cycle Fanconi anemia pathway DNA replication Hippo signaling pathway Axon guidance Regulation of actin cytoskeleton Rap1 signaling pathway Hypertrophic cardiomyopathy (HCM) Proteoglycans in cancer ECM-receptor interaction Steroid biosynthesis PI3K-Akt signaling pathway Arrhythmogenic right ventricular cardiomyopathy (ARVC) Vascular smooth muscle contraction Oocyte meiosis TGF-beta signaling pathway Human papillomavirus infection Dilated cardiomyopathy (DCM)

Table 3 displays the complete list of KEGG pathways identified as significantly enriched (adjusted p-value &lt; 0.05) in the 2D monoculture at 24h compared to 3D monoculture at 24h.

**Table 4:** Pathway enrichment analysis.

2D 72h vs 3D 72h Monoculture	
**Downregulated**	Fluid shear stress and atherosclerosis Proteasome Ether lipid metabolism Ferroptosis IL-17 signaling pathway Glutamatergic synapse Glycolysis/Gluconeogenesis Epstein-Barr virus infection Glycerophospholipid metabolism HIF-1 signaling pathway Central carbon metabolism in cancer
**Upregulated**	DNA replication

Table 4 displays the complete list of KEGG pathways identified as significantly enriched (adjusted p-value &lt; 0.05) in the 2D monoculture at 72h compared to 3D monoculture at 72h.

*Acta2*, *Fap*, and *Ctgf* were selected as canonical fibroblast activation markers to visualize the pancreatic fibroblast state in 2D and 3D cultures. The pancreatic fibroblasts in 3D culture show a significant reduction in the markers *Acta2* and connective tissue growth factor (*Ctgf*) compared to 2D monoculture. In addition, the trend toward further reduction of markers was confirmed over time, suggesting increasing inactivation of pancreatic fibroblasts with prolonged 3D monoculture. *Fap*, a marker of fibroblast activation, is significantly reduced at 72 h in 3D monoculture, but not significantly at 24 h compared to 2D monoculture. Besides these markers, lipid droplet-associated gene expression was also explored. Increased expression of lipid droplet-associated gene *Plin2* is evident in the 3D monoculture sequencing data compared to 2D models at both time points.

### 5.5 Global analysis of KC co-culture and monoculture models

A global comparison of the different co-culture models with KC cells was conducted to identify potential similarities in pancreatic fibroblast responses across the models. For this analysis, all KC co-cultured samples from the two models (2D and 3D) were analyzed together as one group, while all corresponding monocultures were analyzed together as another, the two groups were then compared. Principal component analysis (PCA) was performed to assess the overall variance in gene expression among samples. At 24 h, the first two principal components accounted for 78% (PC1) and 13% (PC2) of the total variance, while at 72 h, PC1 explained 85% and PC2 – 7%. PCA revealed no clear clustering between co-cultured and monoculture samples at the 24-h time point. However, at 72 h, the 2D co-culture samples showed clustering by treatment status. The 3D culture samples showed a greater heterogeneity than the 2D cultures Fig. 9.

**Fig. 9:**
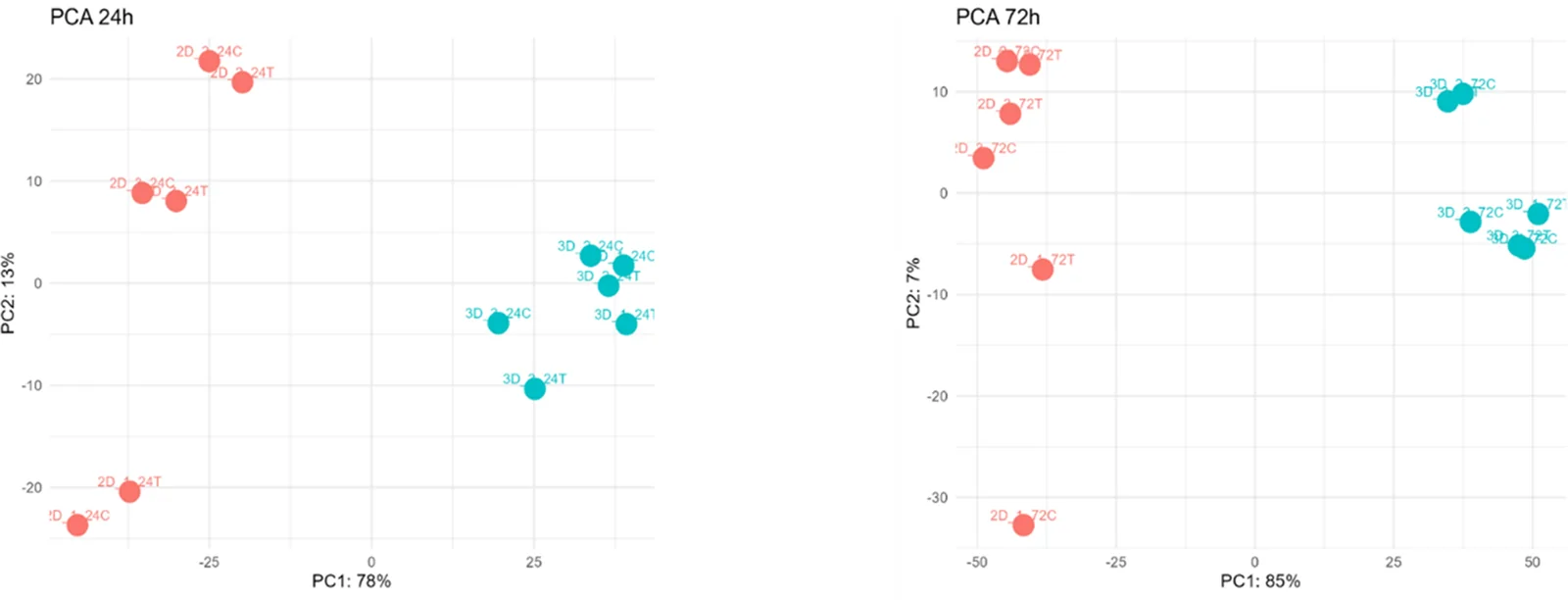
PCAs of the global pancreatic fibroblast-KC co-culture comparison, all co-cultured samples (red, T)/monoculture samples (blue, C) of the KC co-cultures were combined into one group each, naming: C: monoculture, T: co-culture; (A) 24 h time point; (B) 72 h time point.

### 5.6 Influences of KC tumor cells on 3D pancreatic fibroblasts

KC-co-cultured pancreatic fibroblasts were compared to monoculture pancreatic fibroblasts in the two models to understand the transcriptomic effects of tumor cells. Pancreatic fibroblasts co-culture with KCs demonstrated prominently variable gene regulation when comparing co-cultured models to their monocultures Fig. 10. 2D co-culture and monoculture comparison showed 19 DEGs at 24h and 52 at 72h. KEGG pathway enrichment did not have any significant pathway enrichment at his time point. 72h comparison of co-cultured standard pancreatic fibroblasts with monoculture pancreatic fibroblasts showed a significant upregulation of Notch signaling pathway.

**Fig. 10:**
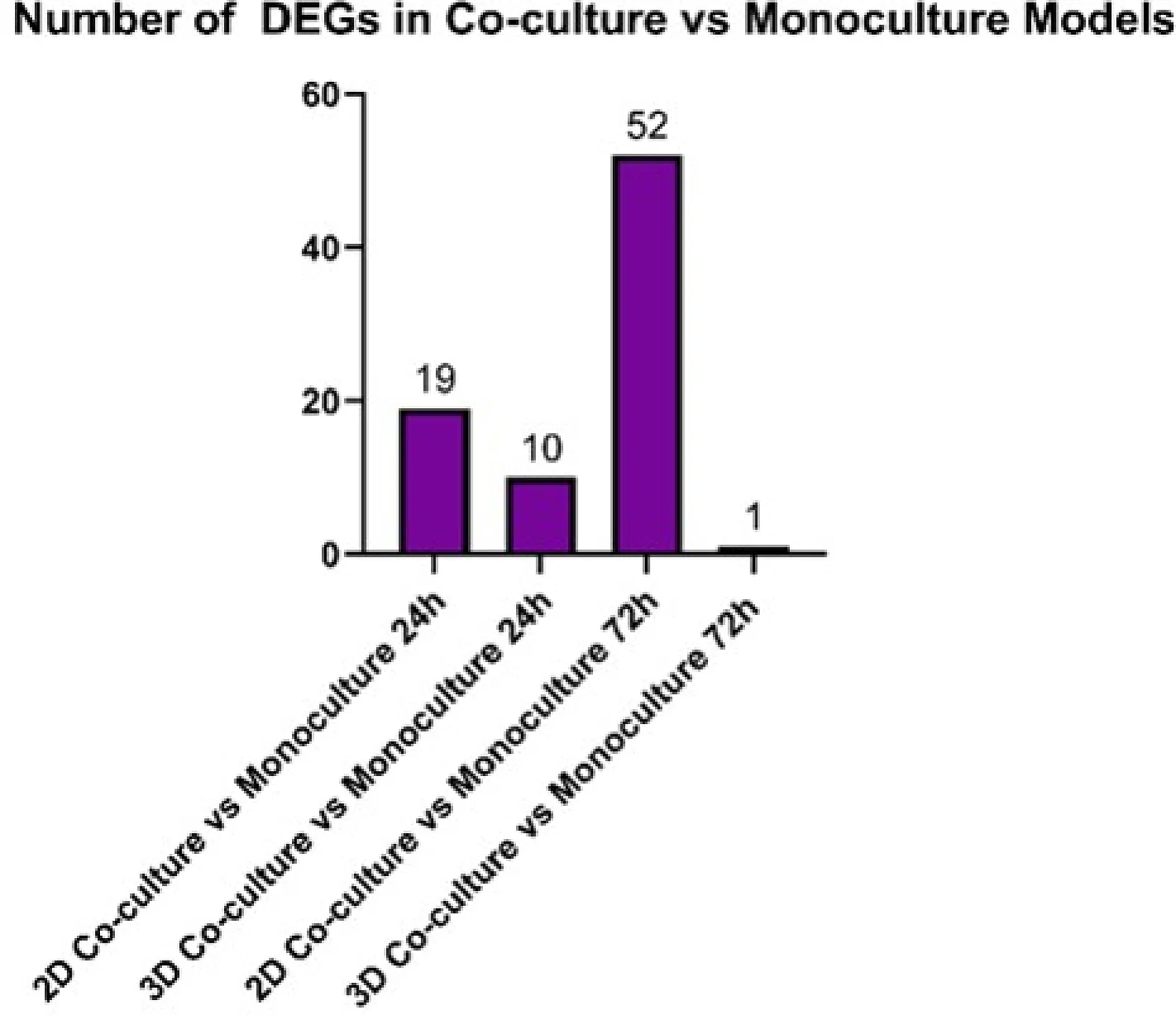
Bar graphs visualizing differential gene expression between KC co-culture and monoculture groups in 24 and 72h data.

In the 3D co-culture models 10 DEGs were present at 24h monoculture comparison and only 1 upregulated gene in 72h co-culture vs monoculture Fig. 11. These include the genes *Arxes2*, *Snap25*, *Cpm*, *Slco4a1*, *Igfbp5*, *Mmp10*, *Mmp13*, *Gm38391*, *Cck*, and *Pi15* at 24 h, and the cytokine *Cxcl1* at 72 h. KEGG pathway enrichment shows 6 significantly regulated pathways in 3D magnetic data at 24h co-culture vs monoculture and 8 at 72h comparison Fig. 12, Table 5, Table 6.

**Fig. 11:**
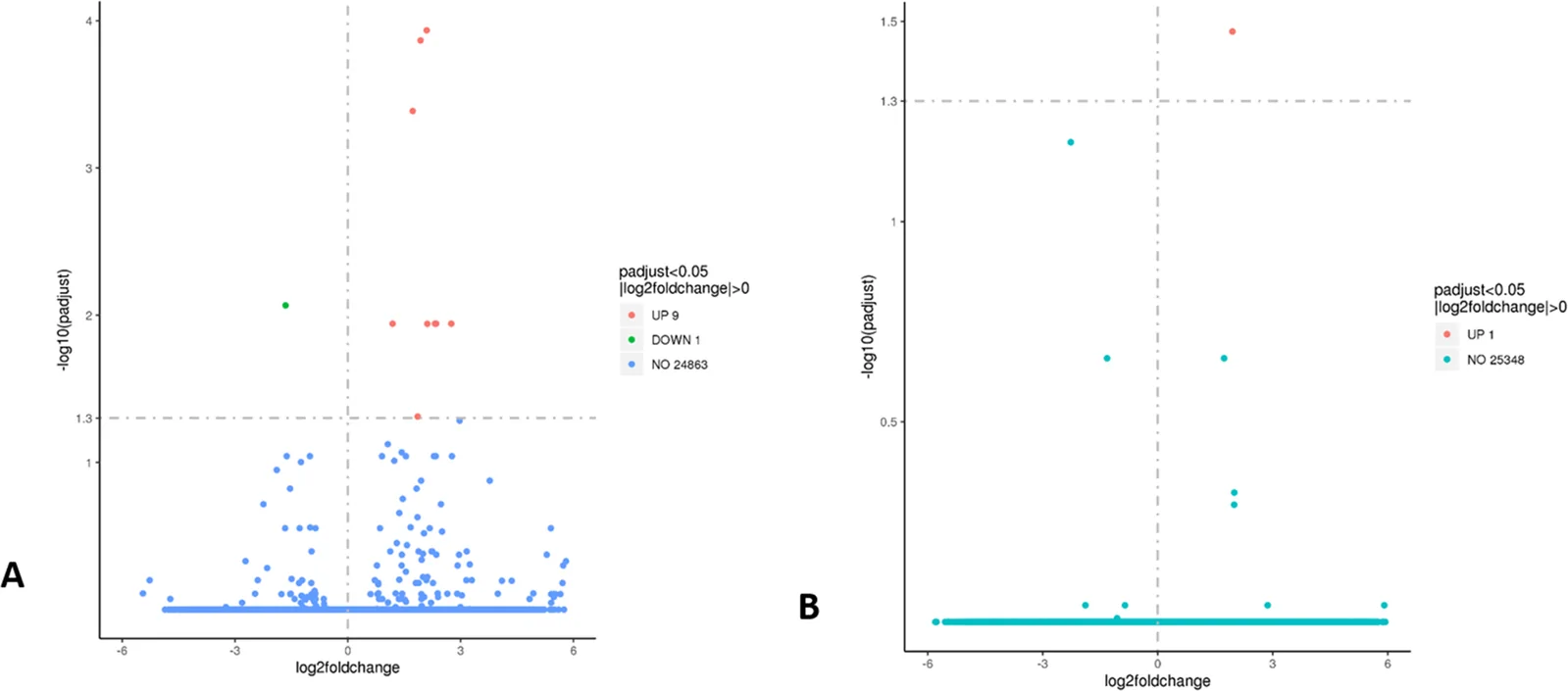
Volcano plots visualizing significant DEGs between 3D co-cultures and monocultures; 10 significantly regulated genes between 24h 3D co-culture and monoculture groups **(A)**; 1 significantly regulated gene between 72h 3D co-culture and monoculture groups **(B)**.

**Fig. 12:**
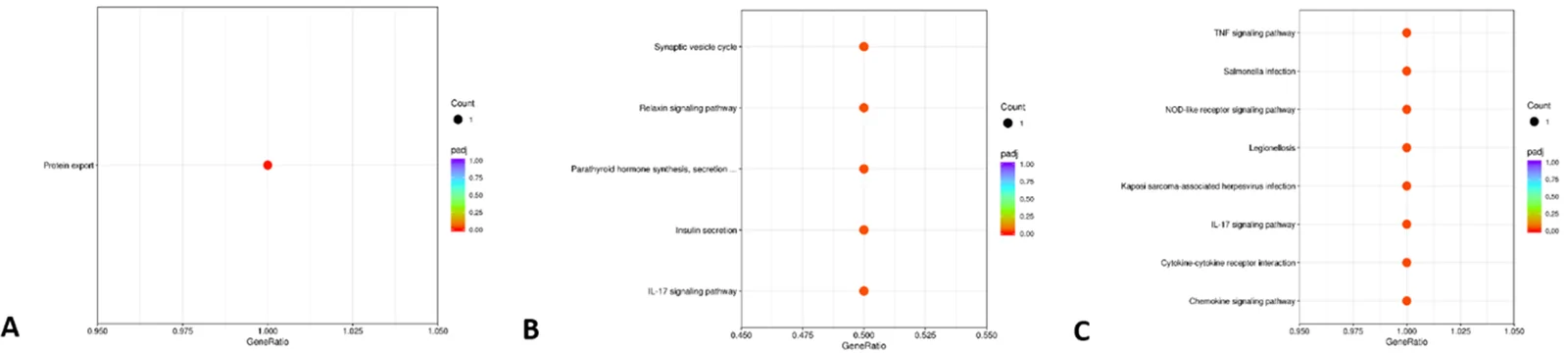
Dot plots for KEGG pathway enrichment demonstrating the regulation between 3D model co-culture and monoculture; 24h down- and upregulated pathways between 3D samples co-culture and monoculture **(A)** and **(B)**; 72h upregulated pathways between 3D co-culture and monoculture samples, so significantly enriched downregulated pathways identified **(C)**.

**Table 5:** Pathway enrichment analysis.

3D 24h Co-culture vs 3D 24h Monoculture	
**Downregulated**	Protein export
**Upregulated**	Synaptic vesicle cycle Insulin secretion IL-17 signaling pathway Parathyroid hormone synthesis, secretion and action Relaxin signaling pathway

Table 5 displays the complete list of KEGG pathways identified as significantly enriched (adjusted p-value &lt; 0.05) in the 3D co-culture at 24h compared to 3D monoculture at 24h.

**Table 6:** Pathway enrichment analysis.

3D 72h Co-culture vs 3D 72h Monoculture	
**Downregulated**	
**Upregulated**	Legionellosis Salmonella infection IL-17 signaling pathway TNF signaling pathway NOD-like receptor signaling pathway Chemokine signaling pathway Kaposi sarcoma-associated herpesvirus infection Cytokine-cytokine receptor interaction

Table 6 displays the complete list of KEGG pathways identified as significantly enriched (adjusted p-value &lt; 0.05) in the 3D co-culture at 72h compared to 3D monoculture at 72h.

To understand the temporal transcriptomic changes happening in co-cultured pancreatic fibroblast and the effects of KC tumor cells, we also compared co-cultured samples between 24 and 72 h 3D co-culture displayed 252 between the co-culture target time points. 563 DEGs between 24h and 72h, while 2D had 6 DEGs. KEGG pathway enrichment in 3D co-culture models demonstrated 9 enriched pathways between 24 and 72 h (Table 7). Similar to the monoculture comparisons, 24h samples showed upregulation of ECM-receptor interaction and focal adhesion pathways. 2D co-culture KEGG enrichment showed three statistically significant pathways (Fig. 13).

**Table 7:** Pathway enrichment analysis.

3D 24h Co-culture vs 3D 72h Co-culture	
**Downregulated**	Hematopoietic cell lineage Steroid biosynthesis
**Upregulated**	ECM-receptor interaction PI3K-Akt signaling pathway Protein digestion and absorption Focal adhesion Human papillomavirus infection Amoebiasis Pathways in cancer

Table 7 displays the complete list of KEGG pathways identified as significantly enriched (adjusted p-value &lt; 0.05) in the 3D co-culture at 24h compared to 3D co-culture at 72h.

**Fig. 13:**
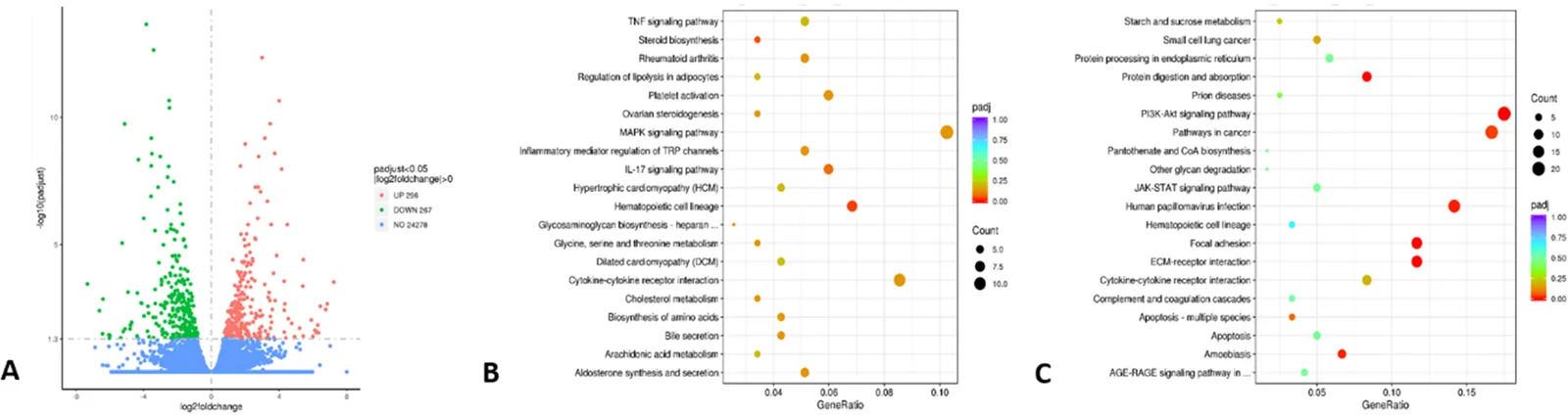
Volcano plot and dot plots visualizing significant gene and pathway regulation between 3D co-cultures between 24 and 72 h; Volcano plot demonstrating 563 significant DEGs between 3D co-culture at 24 and 72h monocultures **(A)**; Dot plots for KEGG pathway enrichment demonstrating the significantly downregulated **(B)** and upregulated **(C)** pathways between 3D samples co-cultures between 24 and 72 h h.

Using known gene panels of iCAF-, myCAF- [11] and apCAF-associated genes [39], the expression of these potential markers in the co-culture samples was also compared between 24 h and 72 h. No clear trend was displayed in the samples with regard to myCAF and apCAF markers. However, the iCAF markers were enriched when comparing 24h co-culture to 72h co-culture in 3D model. Some genes are upregulated at the 72 h time point (*Ccl2*, *Il6*, *Il11*, *Lif*, *Cxcl1*, *Cxcl2*), while others are expressed more strongly at the 24 h time point (*Ndufa4l2* and *Pnpla3*). Exploring the monoculture samples for expression of these iCAF genes over time shows no clear clustering with regard to the 24 h and 72 h time points (Fig. 14).

**Fig. 14:**
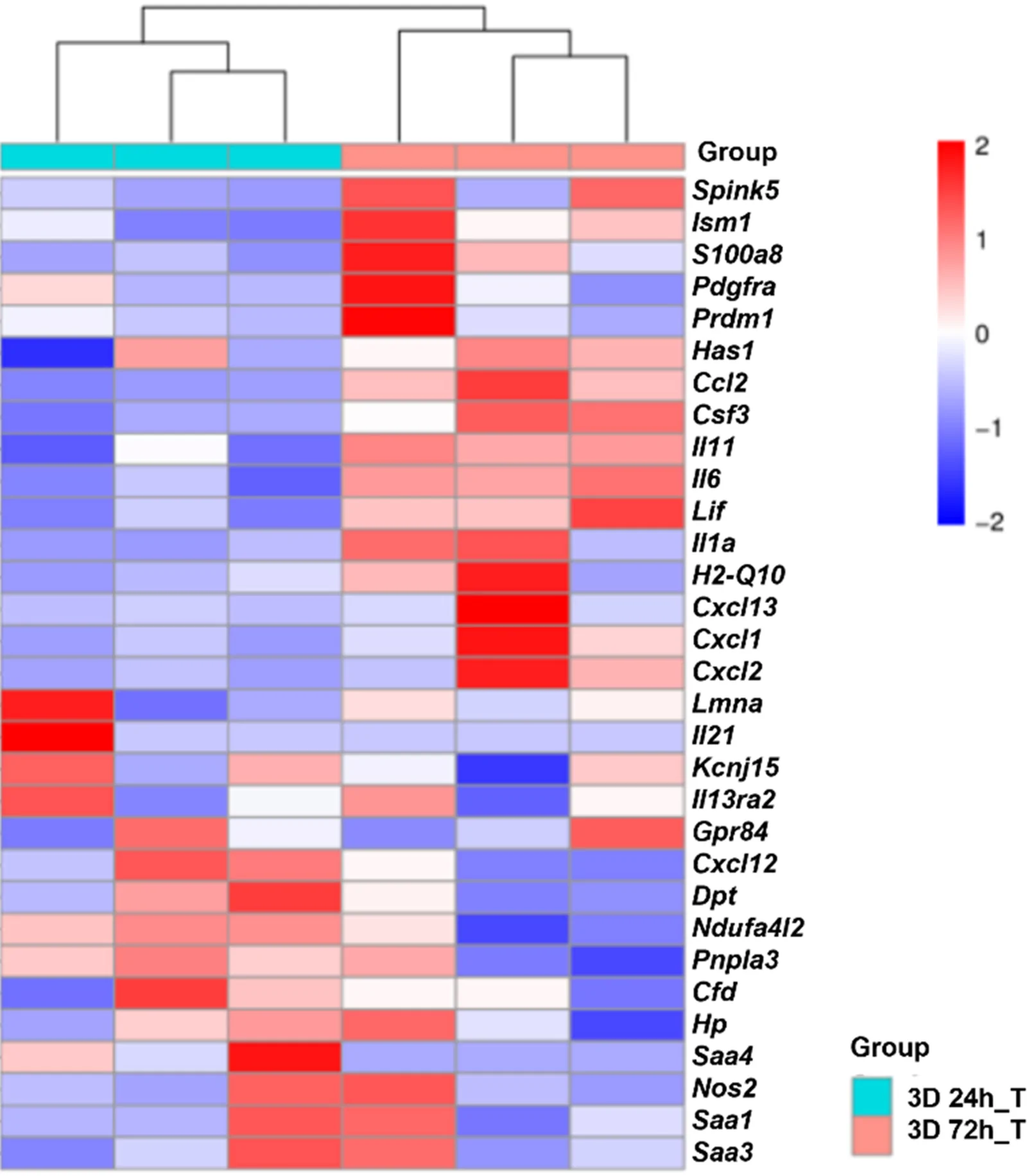
Heatmap with expression of iCAF-associated genes (adapted from Ref. [11]) over time. 3D co-culture samples at 24 h (3D 24_T) and 72 h (3D 72_T). Color coding (red-blue) corresponds to the respective log2 fold change.

### 5.7 Quantitative RT-PCR

To validate the results of the RNA sequencing data and above-described analysis, four target genes were selected and used for qPCR experiments for the 72 h time point. The genes *Acta2*, *Fap*, and *Ctgf* were found to be significantly upregulated in both 2D mono- and co-cultures compared to 3D samples, while PLIN2 was downregulated. Minimal intragroup various in each co-culture model was present, but the trends were prominent and stable. The variation between monoculture KC-exposed and monoculture samples was insignificant. Separate analysis between the 3D mono- and co-culture groups identified a (non-significant) trend towards upregulation of *Acta2* and *Ctgf* in 3D model with KC co-culture (Fig. 15).

**Fig. 15:**
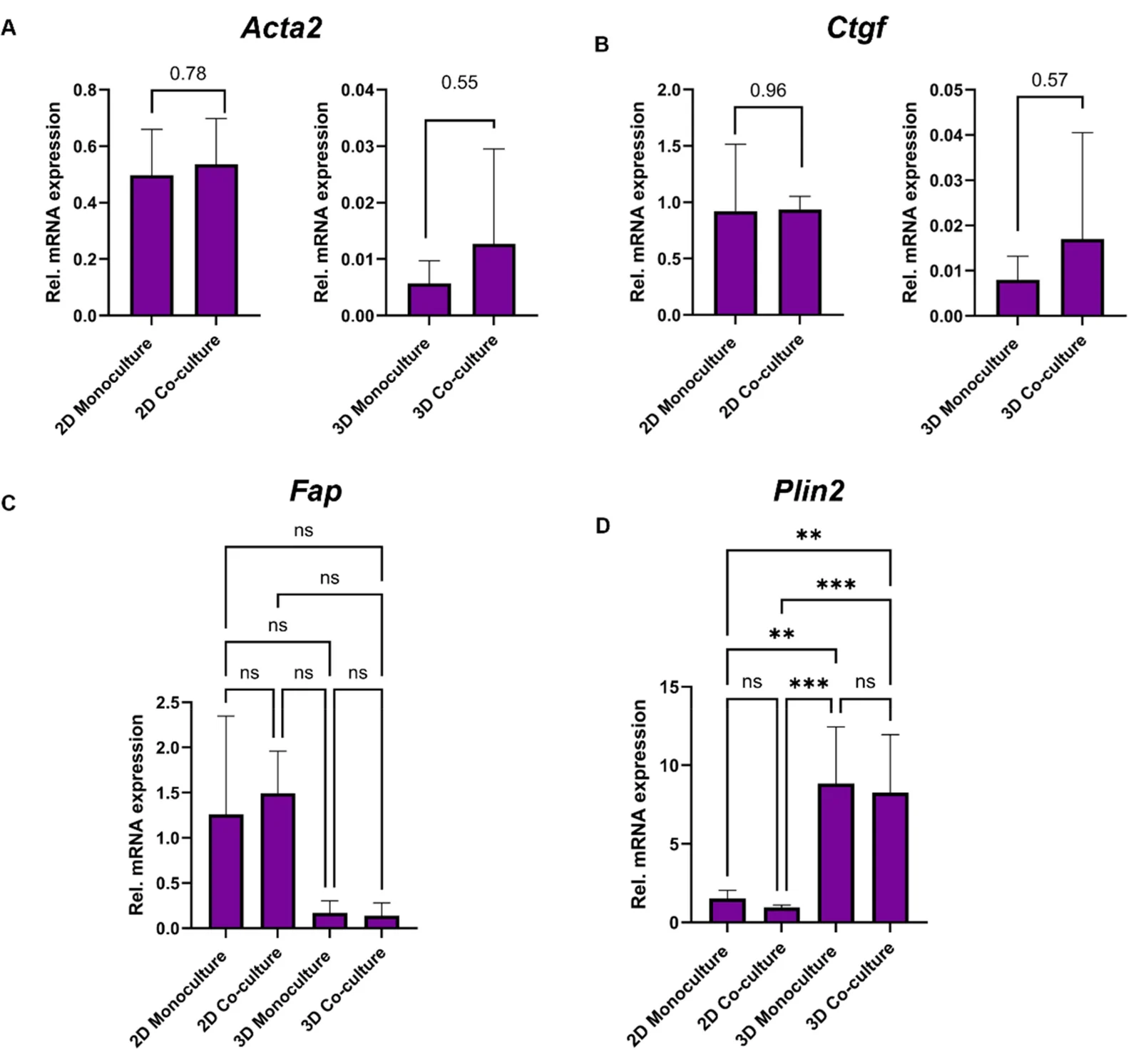
qPCR relative gene expression for RNA sequencing targets, 72h time point; **(A)**, **(B):** Downregulation of *Acta2* and *Ctgf*, in 3D spheroids in both monoculture and KC-exposed samples compared to pancreatic fibroblasts from both 2D mono- and co-cultures; Non-padj significant upregulation of activation markers in 3D co-culture compared to 3D monoculture; **(C)**: Downregulation of *Fap* in 3D spheroids in both monoculture and KC-exposed samples compared to pancreatic fibroblasts from both 2D mono- and co-cultures; **(D)**: Upregulation of *Plin2* in spheroids relative to 2D pancreatic fibroblasts in both mono- and co-culture. The samples in triplicates were normalized to WT 72h pancreatic fibroblast monoculture sample; mean (n = 3) ± SD.

### 5.8 Oil red O staining

Oil Red O staining using 2D and 3D pancreatic fibroblasts spheroids was performed to further investigate the transcriptomic data showing Plin2 gene upregulation in 3D spheroids (Fig. 16). Plin2 upregulation correlates with lipid droplet accumulation can be confirmed by positive Oil Red O stainings. The pancreatic fibroblasts in 2D culture showed mild lipid droplet accumulation in the cytoplasm. The staining of magnetic spheroids in live staining showed distorted visualization explained by the thickness of the spheroid and the magnetic nanoparticles, while staining after frozen section showed prominent development of lipid droplets throught the layers, visualization only partially distorted by the magnetic nanoparticles. Lipid droplet accumulation detected by Oil Red O staining is a characteristic feature of pancreatic stellate cells (PSCs) and the results of this staining could be attributed to PSCs that were isolated among the total fibroblast populations. The SGBS preadipocyte cell line was used as a positive control for the stain which large lipid droplets in adipocyte differentiated cells.

**Fig. 16:**
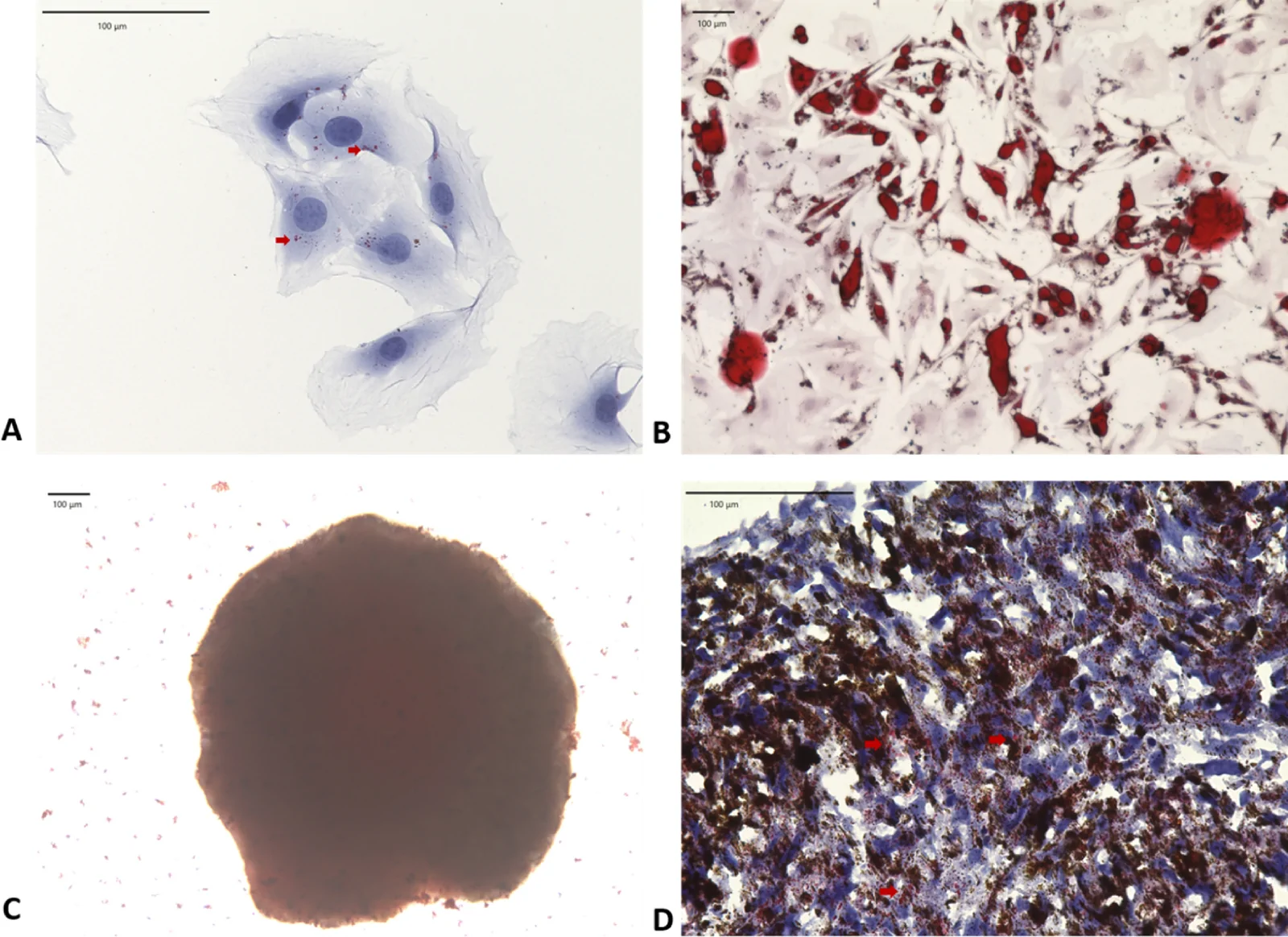
Oil Red O staining supporting the activated state of pancreatic fibroblasts in 2D culture and inactivation of pancreatic fibroblasts in 3D magnetic spheroids. **(A)** Oil Red O staining of pancreatic fibroblasts in 2D culture showing limited lipid droplets **(B)** Oil Red O positive SGBS cells **(C)** 3D magnetic spheroid after Oil Red O staining **(D)** Oil Red O positive staining after spheroid frozen sectioning; Lipid droplets are indicated by red arrows.

## 6 Discussion

Our study demonstrated a prominent variability between pancreatic fibroblasts in monolayer and 3D spheroid cultures. The research model suggested inactivation of pancreatic fibroblasts in spheroids as compared to monolayers shown by downregulation of activation markers Acta2, Fap, and Ctgf. Fibroblast activation and inactivation state is also reflected by lipid metabolism. PLIN2 (Perilipin 2) specifically is a key lipid droplet (LD)-associated protein has been implicated in metabolism of pancreatic adenocarcinoma and pancreatic fibroblasts. It contributes to lipid droplet stabilization and maintenance, especially in stressful metabolic states like hypoxia [40], [41]. Plin2 was interestingly upregulated in both 3D mono- and co-cultures indicating increased lipid accumulation, which was further supported by positive Oil Red O staining indicating a functional shift between the activated and inactivated states in fibroblasts. The combination of activation marker downregulation and upregulation of lipid droplet expression suggested inactivation of fibroblasts in the magnetic spheroids. Since these findings were observed both in mono- and co-cultured spheroids, we concluded that this represented a cell-intrinsic effect as opposed to tumor cell influence. This was not the first study to demonstrate stromal cell inactivation in spheroids. A study by Tseng et al. [42] utilized magnetic levitation for a co-culture model using fibroblast-like aortic valvular interstitial cells (VICs) and endothelial cells for a direct co-culture. The VICs in 3D mono- and co-cultures demonstrated significantly less *Acta2* enrichment, compared to 2D VIC cultures. ECM genes such as *Cola1a1*, *Fn* and *P4h* were also reduced, further indicating the quiescent state of magnetically levitated VICs. The interstitial cell inactivation was partly overcome through direct co-culture with endothelial cells, but not significantly as compared to monolayers.

The variable differential gene expression and pathway enrichment in KC co-culture models displayed the heterogeneous and mode-dependent effects. In the 3D co-culture model, only a small number of genes were significantly regulated in KC-exposed fibroblasts compared to their respective monocultures. Increased expression of matrix metalloproteinases 10 and 13 in KC co-culture of spheroids compared to monocultures suggest increased ECM remodeling at 24 h; while *Cxcl1*, the only upregulated gene in 72h co-culture compared to monoculture, indicated a stronger tumor-stroma crosstalk and CAF activation. While tumor-fibroblast co-culture studies demonstrate fibroblast activation, this is typically achieved by direct co-culture yielding prominent effects on fibroblasts as well as tumor cells through mutual interactions. A study by Norberg et al. [43] established spheroids through direct co-culture using human pancreatic cancer cell lines and mouse pancreatic stellate cells, as well as monocultures for both cell types. The authors observed activation of mouse fibroblasts after tumor cell co-culture by higher *Acta2*, *Fn*, *Col1a1*, and *Tgf* beta gene expression compared to monocultures. The results also demonstrated increased proliferation and a mesenchymal shift in tumor cells, further highlighting a mutual influence in direct co-culture. There are several supporting factors possibly explaining the limited differential gene expression. Our previous project [29] also demonstrated more prominent activation of fibroblasts by tumor cells in closer proximity during inverse co-culture as compared to standard indirect co-culture with further distance between the cells. In addition to the direct and indirect co-culture effects of tumor cells, culture models also affect the paracrine interactions. Fibroblasts in 3D culture, even without tumor cell influence, show significant heterogeneity in the multicellular spheroid. The structure appears to be layered, similar to the previously described tumor spheroids with a necrotic core surrounded by a quiescent zone and an outer proliferative layer. Moderate to larger spheroids (>500 μm) are likely forming two opposing gradients, an increasing gradient of waste products and metabolized towards the center and a decreasing gradient of oxygen, nutrients, and growth factors also towards the center, both supporting to the central necrotic core hypothesis [44]. This is something we also observed in our model as the 3D culture at both time points showed significant enrichment of HIF-1 signaling pathway, a key response of low oxygen [45]. The greater heterogeneity as compared to 2D cultures may obscure somewhat subtle transcriptional changes caused by KC treatment. Autocrine signaling is also an important consideration as intrinsic cellular processes may have stronger impact on pancreatic fibroblasts gene expression compared to paracrine signals from tumor cells further masking the tumor cell influence at least in short term co-cultures. As RNA-seq data demonstrated, 3D culture itself induces major transcriptomic changes over time, in comparison to 2D model which suggests that 3D culture strongly shapes fibroblast behavior and overwhelms the tumor cell-derived factor influence. Despite these limitations some effects from tumor cells were detectable, albeit less pronounced than in 2D co-culture models.

It is hypothesized that in carrier-free 3D culture, fibroblasts initially form loose cell aggregates, which gradually become more compact through the formation of cell-cell contacts, ECM production, and ECM-cell interactions [6]. The observed spheroid formation is consistent with behaviors observed in other cell types cultured in 3D spheroids, likely mediated by cell-cell adhesion proteins and facilitated by the magnetic field [6]. The secretion of extracellular matrix (ECM), previously described in this system [31], aligns with the presence of cell clusters visually confirmed by light microscopy. The consistent upregulation of ECM-receptor interaction in spheroid mono- and co-cultures over time suggests contribution to ECM receptor signaling, especially through integrin genes. In other multicellular spheroids, such as hepatocellular carcinoma models, similar processes have been described, including upregulation of integrins followed by cadherin-mediated adhesion [46]. Cell adhesion and ECM interaction was affected in the 3D culture models, although the changes were heterogeneous. KEGG pathway analysis showed ECM-receptor interaction enrichment at 24h and Rap1 signaling pathways enrichment at 72h. Rap1 is involved in cell adhesion regulation, including integrin and cadherin interactions. In 3D-cultured fibroblasts, both cadherins and integrins were differentially expressed, with some enriched at 24 h and others at 72 h, indicating no clear predominance at either time.

## 7 Conclusion

Our study demonstrated fibroblast inactivation in 3D culture, as evidenced by the downregulation of key activation markers, including *Acta2*, *Ctgf*, and *Fap*, and the upregulation of *Plin2*. This model may therefore provide a useful platform for studying fibroblasts in both activated and inactivated states. Further studies are needed to elucidate the specific role of magnetic levitation in fibroblast inactivation and to determine whether similar effects are observed in other 3D fibroblast culture systems. It would also be of interest to investigate the effects of fibroblasts on cancer cells and to compare paracrine fibroblast–tumor cell interactions between 2D and 3D culture systems.

## CRediT authorship contribution statement

**Nino Javakhishvili** – Methodology, Software, Validation, Formal analysis, Investigation, Data Curation, Writing – Original Draft, Visualization, Funding acquisition.

**Anais Zourelidis** – Conceptualization, Methodology, Software, Validation, Investigation, Visualization.

**Bogusz Trojanowicz** – Conceptualization, Methodology, Validation, Resources, Supervision.

**Yoshiaki Sunami** – Methodology, Validation, Resources, Writing – Review & Editing, Supervision.

**Jörg Kleeff** – Conceptualization, Methodology, Validation, Resources, Writing – Review & Editing, Supervision, Project administration.

## Ethics declaration

This study was conducted in accordance with the ARRIVE (Animal Research: Reporting of In Vivo Experiments) guidelines as well as the following guidelines for animal welfare and/or reporting: European Parliament 2010/63/EU. This study was approved by the Martin-Luther University Halle-Wittenberg.

## Funding

This research (grant number PHDF-21-9027) has been partially supported by 10.13039/501100004801Shota Rustaveli National Science Foundation of Georgia (SRNSFG) to (N.J.). Open access funding was enabled and organized by Projekt DEAL.

## Declaration of competing interest

The authors declare that they have no known competing financial interests or personal relationships that could have appeared to influence the work reported in this paper.

## Data Availability

The bulk RNA sequencing data have been deposited in NCBI's Gene Expression Omnibus [1] and are accessible through GEO Series accession number GSE328041 (https://www.ncbi.nlm.nih.gov/geo/query/acc.cgi?acc=GSE328041).
