# Supplementary material for: Three-dimensional culture suppresses pancreatic fibroblast activation and limits paracrine signaling between pancreatic cancer cells and fibroblasts
Source: Biochem Biophys Rep. 2026 Oct 27;48:102843. doi: 10.1016/j.bbrep.2026.102843 (PMC13734846; doi:10.1016/j.bbrep.2026.102843)
Supplement: Supplementary file 1 [file mmc1.docx]

**Figure FS1** Volcano plot visualizing DEGs between control fibroblasts and fibroblasts exposed to magnetic nanoparticles. No significant DEGs were identified between the cultures, indicating a lack of relevant effects of magnetic nanoparticle exposure to the transcriptome. Of note, the depicted p-values represent non-adjusted p-values.
